# Effectiveness of a community based intervention to delay early marriage, early pregnancy and improve school retention among adolescents in India

**DOI:** 10.1186/s12889-018-5586-3

**Published:** 2018-06-14

**Authors:** Devika Mehra, Archana Sarkar, Priyanka Sreenath, Jagannath Behera, Sunil Mehra

**Affiliations:** 1 Division of Social Medicine and Global Health, Department of Clinical Science, Lund University, Jan Waldenströms gata 35, 205 02 Malmö, Sweden; 2 MAMTA Health Institute for Mother and Child, B-5 Greater Kailash Enclave II, New Delhi, 110048 India

**Keywords:** Early marriage, Contraception, School retention, Pregnancy, Young people

## Abstract

**Background:**

Child marriage is being increasingly recognized globally as a fundamental violation of human rights. Child marriages occur globally in varying degrees across countries and regions. South Asia alone accounted for almost half of the total number of child marriages that have occurred globally. Early marriage can lead to serious ramifications such as school drop-out, early pregnancy, maternal morbidity and mortality. The aim of this study was to assess impact of a multi-pronged community based intervention on early marriage, early pregnancy and school retention among young people in two states of India.

**Method:**

Cross-sectional (post-test) was adopted to assess the effect of the intervention. Multi-stage sampling was adopted for the selection of a sample group of young people aged 10–24 years. A total of 1770 respondents participated in the survey, out of which 826 were males, and 944 were females. The assessment was conducted in eight districts in each of the two states. Descriptive statistics, cross-tabulation, chi square and logistic regression methods were used to analyse the data.

**Results:**

Youth information centres (YIC) as an intervention strategy showed a significant effect towards decrease in the number of early marriages (*Adjusted Odd Ratios [Adj]* 2.25, CI 1.28–3.94), of early pregnancies (Adj 3.00, CI 1.06–8.43) and increase in the number of school retentions (Adj 2.96, CI 2.02–4.34). Access to mass media was also associated with reduction in likelihood of early marriages (Adj 1.79, CI 1.15–2.78), and increase in the number of school retentions (Adj 1.49, CI 1.12–1.97). We also found that there was an increase in mean age of marriage (1.2 years), of conception (.85 years) and in the mean years of schooling (1.54 years) among youth surveyed compared to their older siblings.

**Conclusion:**

Intervention strategies such as YIC and exposure to mass media, showed an effect in reducing early marriage, early pregnancy and improved school retention. Peer education conducted through the YIC proved to be an effective model. Therefore, this multi-component community based intervention can be a potential model for reducing the number of early marriages and its related consequences in other districts of India with similar socio-economic and cultural settings.

## Background

National and international communities are increasingly recognising early marriage (i.e. before the age of 18) as a serious problem in developing countries. Early marriage is not only a violation of human rights but also as a hindrance to national development [1]. Although progress has been made in reducing the prevalence of early marriages, it still remains a pervasive problem in South Asia with females being disproportionately at risk [2]. There is evidence that nearly 50% of all early marriages occur in this region [3]. The prevalence of early marriages in Bangladesh is 64% of all marriages, in India it is 47% and in Nepal 41% [4]. There is empirical evidence from a recent study conducted in these countries that there has been a decline in marriages among girls in early adolescence (below 14 years), but no improvement in the older adolescent age group of 14–17 years [5].

There are multiple drivers of early marriages in India, such as poverty, lack of education, dowry and patriarchal gender norms. There are a wide range of studies from India that show adverse health outcomes on child-brides; studies show that there is an increased likelihood of babies who are stunted or underweight, along with possibilities of miscarriages and still births. There is a lesser likelihood of contraceptive use, delay of first pregnancies and institutional deliveries among women in the age group of 20–24 years [6, 7]. Early marriage can lead to repeated child birth within less than 24-month intervals, multiple unwanted pregnancies, pregnancy terminations and sterilisation at an early age due to lack of access to modern contraceptives [8]. These adverse health outcomes are attributed to an array of factors such as limited access to health information and services, gender power imbalances, poor communication between couples and financial dependence [9]. Early marriages have a higher risk of intimate partner violence, HIV/STIs, maternal morbidity, mortality and depression.

India is a signatory to the Child Rights Convention which recognizes marriages below the age of 18 years as a violation of child rights. The national response to early marriage is well reflected in the legislations, policies, programmes and schemes at the sub-national and district levels. The Early Marriage Act of 2006 has addressed the earlier loopholes to some extent and has a potential to impact the practice of child and early marriage, if executed well [10]. Moreover, India is an active member of the South Asian Initiative to End Violence against Children (SAIEVAC), an apex body of SAARC (South Asian Association for Regional Cooperation), which adopted the ‘Regional Plan of Action to End Child Marriage (2015–2018)’. However, in spite of the strong legal and policy framework, a significant proportion of early marriages are still happening. This reflects the need for multi-pronged strategic interventions to address the complex issue of child and early marriages. Although, there is limited evidence to support scaling-up of effective interventions in India.

The key sectors that can have a direct influence on early marriage are education, health and women’s empowerment, which have still not been able to reach the most vulnerable children and their families. Dropping out of school is a tendency seen in approximately 41% of boys and 37% of girls until grade VIII, with fewer opportunities for vocational education or employment [11]. On the other hand, the reproductive health programmes are not reaching the married adolescents regarding information and education about the use of modern contraceptives. The existing data shows that only 48.5% of the women are using modern contraceptives, resulting in a significant percentage (16%) of girls between 15 and 19 years having begun child bearing [12].

Considering the magnitude of the problem and its impact on the health and social conditions of the younger population of the country, a comprehensive community-based multi-country intervention was planned to improve the sexual and reproductive health of young people by increasing age at marriage, delay of early pregnancy and increased school retention among boys and girls in 18 districts of India, Nepal and Bangladesh. It was a five-year project (2009–2013) funded by the European Union. This study focuses on the evaluation of the intervention conducted in India.

India, as noted above, has a high prevalence of early marriage, which is the prime focus of this study. Uttar Pradesh (UP) and Bihar were chosen as the key states for this intervention due to the high prevalence of early marriages. According to the DLHS III, 2007–2008 (District Level Household Survey), there was a high proportion of girls getting married below the legal age (18 years); 33 and 46% in UP and Bihar respectively [13, 14]. The proportion of boys getting married below the legal age (21 years) was also high, 43.3% in UP and 42.8% in Bihar [13, 14].

Despite the situation there are limited comprehensive interventions in these geographical locations from which we can yield lessons on what works, and even fewer that have been evaluated. Therefore, the aim of this study was to assess if the multi-pronged intervention has had an effect on reducing early marriage, early pregnancy and school retention amongst young people in UP and Bihar.

At a policy level, work on early marriages in India through this intervention, addresses SDG (Sustainable Development Goals) such as poverty reduction, promotion of gender equality, prevention of maternal and child morbidities and mortalities, protection of child rights, as well as the fight against HIV and AIDS [15, 16]. All these challenges are inter-connected through cause and effect relationships and perpetuate early marriage and early child-bearing in the country.

### Project description

The project ‘Improving Sexual and Reproductive Health of Young People by increasing the Age at Marriage and Delaying the First Pregnancy’ was carried out from 2009 to 2013 in 18 sites across three countries— Bangladesh, India and Nepal—with funding from the European Union. In India, the project was set in eight rural sites in high child-marriage prevalence districts in each of the states of Uttar Pradesh and Bihar with a total coverage population of around 100,000 at each site. The intervention was multi-pronged, aiming for three key outcomes—increasing the minimum age of marriage, delaying the first pregnancy and increasing years of schooling as a pathway to delaying marriages. The primary beneficiaries of the project were young people between 10 and 24 years of age, focusing on the most marginalized communities, directly reaching out to 2500–3000 young girls and boys at every site. The strategies included empowerment of young people, community mobilization, advocacy, capacity enhancement of department functionaries involving CSOs (civil society organisations), NGO networks and local media.

The intervention followed the ecological framework reaching out with focused interventions with identified young people, parents, parents-in-law, key community stakeholders including religious leaders, opinion makers in the villages, and elected members from local governance. On the other side, the project worked with district and local administration, relevant departments including Education, Health, Women and Child Development, Panchayati Raj i.e. a local village level assembly and law- enforcement agencies. It prioritized the issue of child marriage for sectoral actions to address the needs and concerns of both unmarried and married young people.

The intervention package included age and culturally appropriate life skill-based educational sessions, focusing on SRHR (sexual and reproductive health and rights) from gender and sexuality perspectives. The national curriculum ‘Life Skills & Adolescent Education Programme’ was adapted to strengthen the components on early marriage and early pregnancy to suit the objectives of the intervention. The curriculum had additional activities with the understanding to promote education retention as a pathway to delay the age of marriage and first pregnancy.

Young people were identified and brought into a small group with 20–25 members in each of the intervention villages. The selection of one male and one female youth to serve as peer educator was made in consultation with the group members. These peer educators were trained for five days with a curriculum on life skills-based comprehensive education on SRHR. There was a special focus on the importance of completing school education, learning vocational skills, delaying marriage and pregnancy. The behaviour change communication tools were developed after formative research, and pretesting with communities, and were provided to project community workers and peer educators. A series of six posters titled *Babli Kare Sawaal* (Babli*,* is a girl in early adolescence posing pertinent questions to the society at large, without being at risk of hurting community sentiments) in Hindi, along with games and a set of picture cards, that were provided for young people to plan these important events of life.

Additionally, aiming to serve youth from five to six villages, a ‘Youth Information Centre’ (YIC) was set up within a cluster of villages, to facilitate peer communication and learning through entertainment on issues of SRHR. The idea of YIC was in line with the concept of ‘Safe Space’, a safe and supportive environment that informs and motivates young people to make healthy choices [13]. The YIC was a modest structure with minimum functional facilities, housed in spaces provided by the community, mostly in public institutions such as panchayat (translation) buildings, schools or child health care centres, while in some places even in private mud houses, promoting community ownership. The project supplied edutainment material to these YICs. and a small budget to organize monthly activities ensuring the participation of the larger community. YICs also had friendly and sensitive tools to encourage queries and access referrals for counselling and treatment. The peer educators of the village where the YIC was located had the advantage of using the space to conduct sessions with peers. The project established 72 YICs, which were visited by 46,955 young people.

At the family and community level, the project reached out with structured meetings to parents, members of local governance, religious leaders and other key community stakeholders that included teachers and health-service providers. Local civil society groups, non-government organisations and media were sensitized and supported to undertake advocacy, awareness and education campaigns for preventing child and early marriages, through their existing work.

At the system level, the project made an effort to facilitate collective action by various departments to address child and early marriage, by bringing in district administration, law enforcement bodies and relevant departments with programmes and development schemes specifically for adolescents and youth. To boost the efforts for effective enforcement of the ‘Prohibition of Child Marriage Act, 2006’ the project advocated the rolling out and implementation of two other critical efforts by the national government: in-school life-skills based adolescent education (a national programme to provide SRHR education) and adolescent reproductive and sexual health programme (based on Youth Friendly Health Services). Two days of training was followed by refresher sessions of the functionaries in the department. These included teachers, doctors and frontline health workers in intervention villages. These training and refresher sessions were conducted for mentoring support to work with, and for, young people whose focus was on delaying the age of marriage and first pregnancy in their respective communities.

## Method

### Study design and setting

The study design was cross-sectional (post-test study) with a mixed method approach. Multi-stage sampling was adopted for the selection of a sample of young people aged between 10 and 24 years. The first stage was selecting the states for the intervention. The second stage was selection of the districts, and the third stage was selection of villages. The initial survey was conducted in all eight intervention sites across two states of India (Uttar Pradesh and Bihar). The sites were located in the districts of West Champaran, Nalanda, Navada, Vaishali in the state of Bihar, and in Chitrakoot, Gonda, Hardoi, Siddharth Nagar in the state of Uttar Pradesh.

A total of 1770 respondents participated in the survey out of which 826 were males and 944 were females. Five cases were not included in the analysis as they did not state their age. The response rate of the data collection was 99%. All the districts in the project intervention area were covered. Six sites were randomly selected from each district—the number of sites having YICs and those without YICs being roughly equal in number. From the centre of the sampled site, a team of investigators spread out in different directions. In each direction, the team covered consecutive households having young people. One young person was interviewed from each selected household. If the household had more than one young person, then preference for interview was given to the youngest person who belonged to the age group that was proportionately less among the three age groups (10–14, 15–19 and 20–24 years). The young people were interviewed using a structured guide that captured knowledge and practice regarding education, marriage and child bearing, among other questions. Sibling history regarding education, marriage and conception was also collected in consultation with parents of the respondent.

### Measurement tools

In 2009, a baseline study was conducted with quantitative tools for household survey for information on schooling, age of marriage and that of first pregnancy, for young men and women. A ‘Participatory Learning and Action’ method was also adopted to foster partnership and ownership of the project issues in the communities. However, owing to the difference in sampling design and computation of indicators, the baseline and endline findings were not comparable, and hence could not be analysed. Therefore, retrospective estimation of years of schooling, age of marriage and of first pregnancy for the older siblings of respondents at the sample households, was made a part of the quantitative analysis.

Apart from the quantitative data that had been used and analysed, qualitative ethnographic fieldwork was also undertaken with young people, their parents and stakeholders in the government (medical officers and frontline functionaries), the media, NGOs and communities covering a wide range of stakeholders that could affect the outcomes. Focused group discussions and in-depth interviews (IDIs) were conducted with the study participants. A qualitative fieldwork was conducted in an iterative manner to generate evidence about their involvement in the programme and its impact on early marriage, early pregnancy and school retention amongst young people in the community.

### Data collection

The data was collected through a questionnaire which was based on socio-demographic factors, marriage, pregnancy, education and exposure to mass media, YICs and information on youth-friendly health services, among other factors. The research team informed the participants about the purpose of the study and the estimated time they would spend participating in the survey. They were also told that if they were uncomfortable during any stage of the data collection, they could withdraw or not answer that question. This informed consent was taken from young people and stakeholders before the data collection.

Confidentiality of data was maintained at all stages of the study. Consequently, identification numbers were used for the data collected and for conducting the analysis, allowing non-disclosure of their personal identities. Training was imparted to the field team about ways to maintain confidentiality in field settings. Confidentiality during data collection in community-based fieldwork in India can be challenging, with curious family members and neighbours posing as bystanders. Members of the team were trained to ward off such onlookers. The project conformed to the ethical principles of autonomy, confidentiality and non-maleficence. Informed consent was taken from all study participants. Voice recording was also obtained in the case of consenting respondents. Contact details (including address and phone numbers) of the organizations were shared with them so that they could voice any further concerns. Ethical approval for the data collection of this project was taken from the Institutional Review Board at MAMTA Health Institute for Mother and Child. The data was collected by IIHMR (Indian Institute of Health Management and Research). The questionnaire was developed by researchers at IIHMR and at the MAMTA Health Institute for Mother and Child.

### Data analysis

The analysis was done using the Statistical package for social sciences (SPSS) Version 22.0. Stratified analysis was conducted for male and female participants. The prevalence of socio-demographic factors and intervention strategies such as exposure to peer educators/leaders, access to media, to youth information centres, exposure to life-skills education, and information about youth-friendly health centres was calculated in percentages. Binary logistic regression analysis was done to calculate the crude odds ratio (OR) with 95% confidence interval (CI). Socio-demographic factors and intervention strategies were taken as intermediary or confounding factors to assess its association with early marriage, early pregnancy and school retention. Multivariable logistic regression was used to control for confounding by stepwise adjusting for exposure to the intervention’s youth information centres, access to media, peer educators, and socio-demographic factors such as caste and income. The sample was calculated using the design effect along with the rate of non-response.

### Definition of variables

*Age* was categorized into three groups 10–14, 15–19 and 20–24 years based on the different characteristics of this population in relation to the outcomes of the study.

*Marriage* was used as ‘never married’ and ‘ever married’.

*Educational level* was used as ‘less than primary education’ and ‘more than primary education’.

*Income* was dichotomized as less than 3000, and more than 3000, and the cut-off was based on the definition of BPL (below poverty line) in India.

*Caste* was categorized into ‘general’ and another group which was combined with other backward classes, scheduled caste and scheduled tribes (SC/ST).

*Exposure to peer education* was response to the question, ‘Have you ever met a peer educator?’, which was coded as ‘yes’ and ‘no’.

*Access to media* combinedexposure to television, radio and newspapers. All the participants who stated that they had any of the three, were coded as ‘yes’ and the rest were in the ‘no’ category.

*Exposure to Youth Information Centres (YIC)* was a response to the question,‘Did you participate in any of the activities of the YIC’, which was coded as ‘yes’ or ‘no’.

*Exposure to life skills education* was coded as ‘yes’ and ‘no’.

*Information about youth-friendly health services* was categorized as ‘yes’ and ‘no’.

*Sibling variable* was created as our operational definition for siblings; we considered siblings as born to the same parents, including twins, triplets or adopted.

Outcome variables:

- *Age at first marriage* was coded as ‘married before the legal age of marriage’, ‘marriage after the legal age of marriage’ and ‘never married’.
- *Age of conception* was dichotomized with the cut-off point as 19 years. According to the WHO definition of adolescent pregnancy, below 19 years is considered ‘a high risk pregnancy’.
- *Currently going to school* was coded as ‘yes’ and ‘no’.

## Results

### Socio-demographic characteristics

The distribution of socio-demographic factors, intervention strategies, prevalence of early marriage, early pregnancy and school dropouts is seen in Table 1. The participation of males was 46.7% which was less than the participation of females (53.3%). The majority of the participants belonged to the age group of 10–14 years (43.1%). The percentage of participants that were ever married in this population was 21%, with a larger majority (79%) of them not being married. A higher percentage of females had less than primary education (40.7%) compared to boys (36.8%).

**Table 1:** Distribution of socio-demographic factors, intervention strategies, school retention, early marriage and early pregnancy in UP and Bihar (India)

	All *n* = 1770	Male *n* = 826	Female *n* = 944	*P* value
*Age*				
10–14	763 (43.1)	375 (45.2)	390 (41.3)	.245
15–19	501 (28.3)	223 (27.0)	278 (29.4)	
20–24	506 (28.3)	230 (27.8)	278 (29.2)	
*Marital status*				
Never married	1399 (79.0)	718 (86.9)	681 (72.1)	.000
Ever married	371 (21.0)	108 (13.1)	263 (27.9)	
*Educational level*				
> Primary	994 (61.2)	504 (61.0)	490 (59.3)	.114
≤ Primary	629 (38.8)	293 (36.8)	336 (40.7)	
*Income*				
> 3000	1468 (82.7)	622 (75.3)	841 (89.1)	.000
≤ 3000	307 (17.3)	204 (24.7)	103 (10.9)	
*Caste*				.024
General	413 (23.3)	213 (25.8)	200 (21.2)	
Other backward classes/Scheduled class/Scheduled tribes	1357 (76.7)	613 (74.2)	744 (78.8)	
*Exposure to peer educators*				
Yes	250 (15.4)	62 (8.4)	188 (21.3)	.000
No	1373 (84.6)	680 (91.6)	693 (78.7)	
*Access to Media*				
Yes	1305 (73.7)	508 (61.5)	797 (84.4)	.000
No	465 (26.3)	318 (38.5)	147 (15.6)	
*Exposure to Youth Information Centre*				
Yes	378 (21.4)	170 (20.6)	208 (22.0)	.457
No	1392 (78.6)	656 (79.4)	736 (78.0)	
*Age at marriage* *				
Married after the legal age of marriage and never married	1497 (89.6)	794 (98.0)	703 (81.7)	.000
Married before the legal age at marriage	173 (10.4)	16 (2.0)	157 (18.3)	
*Age of conception*				
> 19 years	253 (68.4)	85 (78.7)	168 (64.1)	.007
≤ 19 years	117 (31.6)	23 (21.3)	94 (35.9)	
*Currently going to school*				
Yes	1188 (73.2)	601 (75.3)	587 (71.1)	.053
No	436 (26.8)	197 (24.7)	239 (28.9)	
*Exposure to life skills education*				
Yes	400 (35.2)	132 (23.2)	268 (47.1)	.000
No	737 (64.8)	436 (76.8)	301 (52.9)	
*Information about youth friendly health services*				
Yes	395 (35.4)	167 (29.9)	228 (40.8)	.000
No	722 (64.6)	391 (70.1)	331 (59.2)	

^*^ Caste which were married before the intervention have been excluded from the analysis and legal age of marriage in India is 18 years for girls and 21 years for boys

The majority of the population self-reported that they came from a SC/ST or from some other backward class (76.7%). Female participants had a higher exposure to peer education (21.3%) compared to males (8.4%). Access to media was also higher in females (84.4%) compared to males (61.5%). Response to exposure to Youth Information Centres was 21.4% in our sample. There was a much higher prevalence of girls who were married before the legal age of marriage (18.3%) compared to boys (2%). In our sample, 31.6% of the females stated that they conceived a child before 19 years of age and 21.3% males responded that their partners did so before 19 years. Nearly 27 % of the youth of our sample was currently not going to school. A higher percentage of females (47.1%) compared to males (23.2%) have had exposure to life skills education. The percentage of participants who had information about youth friendly health services in our sample was 35.4%.

### Association to early marriage

Table 2 presents the binary logistic regression analysis between the predictor variables (intervention strategies, socio-demographic variables) and outcome variable of early marriage. The results highlight that participants not going to school then had a much higher likelihood of getting married before attaining 18 years: 7.77 (5.29–11.40). There were marked gender differences: females had a much higher likelihood 10.07 (6.50–15.60) compared to males 3.85 (1.38–10.77). A similar pattern was also observed for men however the sample size was low for them (ORcrude3.85, CI 1.38–10.77). We also found that youth belonging to a lower caste had a higher risk of having an early marriage (ORcrude1.82, CI 1.18–2.79) than youth belonging to the general caste/more privileged social groups. In light of the intervention strategies of the project, we found a significant association with the peer educators, but only for females (ORcrude2.69, CI 1.55–4.65). Access to media showed a significant impact on decreasing early marriages (ORcrude2.12, CI 1.38–3.24). Further, our core intervention strategy, exposure to the Youth Information Centres, was significant among females (ORcrude2.71, CI 1.61–4.75) but not males.

**Table 2:** Cross-tabulation and bi-variate analysis of the socio-demographic factors intervention strategies and early marriage in UP and Bihar (India)

	Early Marriage	All		Male		Female
*Currently going to school*						
Yes	44 (3.7)		7 (1.2)		37 (6.4)	
No	88 (23.1)	7.77 (5.29–11.40)*	8 (4.3)	3.85 (1.38–10.77)*	80 (40.6)	10.07 (6.50–15.60)*
*Income*						
> 3000	157 (11.4)		12 (2.0)		145 (18.9)	
≤ 3000	16 (5.4)	.45 (.26–.76)*	4 (2.0)	1.01 (.32–3.17)	12 (12.9)	.64 (.33–1.20)
*Caste*						
General	27 (6.7)		1 (0.5)		26 (13.5)	
OBC/SC/ST	146 (11.5)	1.82 (1.18–2.79)*	15 (2.5)	5.40 (.70–41.09)	131 (19.6)	1.57 (1.00–2.48)
*Exposure to peer educators*						
Yes	18 (7.5)		2 (3.2)		16 (9.0)	
No	144 (11.2)	1.54 (.92–2.58)	13 (2.0)	.60 (.13–2.71)	131 (21.1)	2.69 (1.55–4.65)*
*Access to Media*						
Yes	27 (6.0)		6 (1.9)		21 (15.4)	
No	146 (11.9)	2.12 (1.38–3.24)*	10 (2.0)	1.05 (.38–2.90)	136 (18.8)	1.27 (.77–2.09)
*Exposure to Youth Information Centre*						
Yes	20 (5.4)		2 (1.2)		18 (9.0)	
No	153 (11.8)	2.33 (1.44–3.78)*	14 (2.2)	1.86 (.42–8.28)	139 (21.1)	2.71 (1.61–4.57)*

*P value *p*<0.05

### Association with early pregnancy

Table 3 presents the binary logistic regression analysis between the predictor variables (intervention strategies, socio-demographic variables) and the outcome variable of (early pregnancy. The results show that girls who came from a backward class were more likely to conceive early (ORcrude1.78, CI 1.20–2.62) and we also found that those men who said that their wives came from a backward class had almost twice the likelihood to conceive early (ORcrude1.92, CI 1.28–2.87) as compared to those from the better-off classes. Regarding the intervention strategies, we found that having no access to media was associated with early pregnancy for boys reporting for their wives (ORcrude1.52, CI 1.08–2.14) and girls (ORcrude1.85, CI 1.18–2.90). We also found that young people who did not have exposure to Youth Information Centres had a higher risk for early pregnancy for boys (reporting for their wives) (ORcrude2.89, CI 1.76–4.76) and girls (ORcrude 2.42, CI 1.61–3.62).

**Table 3:** Cross-tabulation and bi-variate analysis of the socio-demographic factors, intervention strategies and early pregnancy in UP and Bihar (India)

	N Pregnancy below 19 years	OR	Male**	OR	Female	OR
*Currently going to school*						
Yes	13 (19.4)		4 (21.1)		9 (18.8)	
No	62 (20.0)	1.69 (.86–3.32)	16 (20.8)	.98 (.28–3.37)	46 (33.6)	2.19 (.98–4.91)
*Income*						
> 3000	103 (32.1)		18 (21.7)		85 (35.7)	
≤ 3000	14 (28.6)	.85 (.44–1.64)	5 (20.0)	1.23 (.85–1.78)	9 (37.5)	.76 (.45–1.27)
*Caste*						
General	12 (21.1)		2 (15.4)		10 (22.7)	
OBC/SC/ST	105 (33.5)	1.89 (.96–3.73)	21 (22.1)	1.92 (1.28–2.87)*	84 (38.5)	1.78 (1.20–2.62)*
*Exposure to peer educators*						
Yes	8 (20.0)		3 (42.9)		5 (15.2)	
No	105 (34.0)	2.06 (.92–4.63)	19 (20.7)	1.41 (.73–2.72)	86 (39.6)	1.44 (.98–2.10)
*Access to Media*						
Yes	20 (27.4)		8 (22.9)		12 (31.6)	
No	97 (32.7)	1.28 (.73–2.27)	15 (20.5)	1.52 (1.08–2.14)*	82 (36.6)	1.85 (1.18–2.90)*
*Exposure to Youth Information Centre*						
Yes	5 (11.9)		1 (7.1)		4 (14.3)	
No	112 (34.1)	3.84 (1.47–10.03)*	22 (23.4)	2.89 (1.76–4.76)*	90 (38.5)	2.42 (1.61–3.62)*

**The response for the males is regarding their wife’s age at first conception

*P value *p*<0.05

### Association with school retention

Table 4 presents the binary logistic regression analysis between the predictor variables (intervention strategies, socio-demographic variables) and the outcome variable school retention. We found that there was an association between coming from a lower caste and not going to school (ORcrude1.86, CI 1.40–.46). This finding was significant for both males and females. In our analysis we found that exposure to peer education did not show an association with school retention. We found that no access to media had a significant association with not going to school (ORcrude1.69, CI 1.30–2.19); this finding was significant for boys and girls. We also found that no exposure to Youth Information Centres was associated with lack of school retention for boys (ORcrude2.89, CI 1.75–4.75) and girls (ORcrude 2.42, CI 1.61–3.62).

**Table 4:** Cross-tabulation and bi-variate analysis of the socio-demographic factors intervention strategies and school retention in UP and Bihar (India)

	Currently not going to school	All		Male		Female
*Income*						
> 3000	361 (26.9)		143 (23.7)		218 (29.5)	
≤ 3000	75 (26.6)	.99 (.74–1.32)	54 (27.7)	1.23 (.85–1.78)	21 (24.1)	.76 (.45–1.27)
*Caste*						
General	74 (18.5)		35 (16.6)		39 (20.5)	
OBC/SC/ST	362 (29.6)	1.86 (1.40–46)*	162 (27.6)	1.91 (1.28–2.87)*	200 (31.4)	1.78 (1.20–2.62)*
*Exposure to peer educators*						
Yes	56 (23.0)		12 (19.7)		44 (24.2)	
No	358 (28.4)	1.33 (.96–1.83)	170 (25.7)	1.41 (.73–2.72)	188 (31.4)	1.44 (.98–2.10)
*Access to Media*						
Yes	91 (19.9)		64 (20.1)		27 (19.4)	
No	345 (29.6)	1.69 (1.30–2.19)*	133 (27.7)	1.52 (1.08–2.14)*	212 (30.9)	1.85 (1.18–2.90)*
*Exposure to Youth Information Centre*						
Yes	54 (14.6)		20 (11.9)		34 (16.8)	
No	382 (30.5)	2.57 (1.87–3.50)*	177 (28.1)	2.89 (1.75–4.75)*	205 (32.9)	2.42 (1.61–3.62)*

*P value *p*<0.05

Table 5 shows the regression model that was adjusted for the socio-economic and confounding factors that could be associated with early marriage in our sample. In our adjusted model we found that YICs had a significant association with early marriage (ORadj2.25, CI 1.28–3.94). The effect was statistically significant for females (ORcrude2.08, CI 1.11–3.89) but not for males. We also found an association between no access to mass media and early marriage (ORadj1.79, CI 1.15–2.78) but it did not have an effect on early marriage when stratified for sex. Coming from a lower caste was also found to be associated with early marriage (ORadj1.59, CI 1.02–2.50), in females (ORadj1.64, CI 1.01–2.67) but not in males.

**Table 5:** Association between intervention strategies and early marriage among young people in UP and Bihar

	All	Males	Females
YIC	2.25 (1.28–3.94)*	2.14 (.40–11.43)	2.08 (1.11–3.89)*
Access to mass media	1.79 (1.15–2.78)*	.80 (.27–2.38)	1.05 (.61–1.79)
Peer education	1.03 (.57–2.85)	.44 (.08–2.37)	1.83 (.96–3.49)
Caste	1.59 (1.02–2.50)*	4.70 (.61–36.30)	1.64 (1.01–2.67)*
Income	.44 (.26–.75)	1.08 (.33–3.47)	.67 (.35–1.29)

*P value *p*<0.05

Table 6 shows the multivariate analysis after adjusting for confounders that could be associated with early pregnancy. In the fully adjusted model we found that participants not using the YICs had a higher likelihood of early pregnancy (ORadj3.00, CI 1.06–8.43).

**Table 6:** Association between intervention strategies and early pregnancy among young people in UP and Bihar

	All
YIC	3.00 (1.06–8.43)*
Access to mass media	1.04 (.57–1.92)
Peer education	1.45 (.60–3.49)
Caste	1.77 (.88–3.56)
Income	.77 (.39–.1.50)

*p* value *p*<0.05*

Table 7 represents the multivariate analysis that was adjusted for the socio-economic and other confounding factors that could be associated with school retention. We found an association between access to YICs and school retention, that was significant among males (OR adj 2.72, CI 1.53–4.82) and females (ORadj3.16, CI 1.89–5.29). Access to media was also found to be significant for females (ORadj1.70, CI 1.06–2.74) but not for males. Coming from a lower caste had an association with decreased school retention among males (ORadj1.87, CI 1.20–2.90) and females (ORadj1.73, CI 1.15–2.62). Table 8 shows the current and retrospective mean age of marriage, mean age of conception and mean age of schooling for the respondents and their elder siblings. We found that the mean age of marriage increased from 15.83 years in the older sibling to 17.03 years in the participants after the project ended. The age of conception increased from 17.54 years to 18.39 years. The mean years of schooling increased from 6.39 years to 7.93 years.

**Table 7:** Association between intervention strategies and school retention among young people in UP and Bihar

	All	Males	Females
YIC	2.96 (2.02–4.34)*	2.72 (1.53–4.82)*	3.16 (1.89–5.29)*
Access to mass media	1.49 (1.12–1.97)*	1.25 (.87–1.82)	1.49 (1.12–1.97)*
Peer education	.72 (.49–1.06)	.79 (.38–1.63)	.73 (.45–1.19)
Caste	1.79 (1.32–2.42)*	1.87 (1.20–2.90)*	1.73 (1.15–2.62)*
Income	.90 (.67–.1.22)	1.07 (.72–1.57)	.78 (.45–1.33)

*p* value *p*<0.05

**Table 8:** Current and Retrospective mean age at marriage, mean age of conception and mean age of schooling of the respondents and elder sibling*

	Males	Females	Total
Mean age at marriage	18.63	16.29	17.03
Mean age at marriage of older sibling	17.59	15.37	15.83
Age at first conception	–	18.39	
Age at fist conception of older sibling		17.54	
Mean years of schooling	7.95	7.81	7.93
Mean years of schooling of older sibling	6.72	6.19	6.39

*The above figures are obtained from the endline reports of this project

## Discussion

There is high prevalence of early marriage, poverty and low education in India, especially in Uttar Pradesh and Bihar. The evidence revealed that adolescent girls who were not going to school were ten times more likely to have an early marriage, whereas boys had a three times higher likelihood for the same. This finding is in cognizance with literature from other studies [17, 18]. The data revealed that females who did not have exposure to the peer educators had a higher risk of marrying early. However, the effect of this association was not significant in the regression model, which could be due to other confounding factors.

One of the major findings of this study showed that access to YICs, which was one of the core intervention strategies of the project, led to a reduction of early marriage among girls. The peer educators conducted the sessions with young people at these YICs, which provided a safe and supportive environment and motivated young people to make healthy choices [19]. These YICs also provided young people with opportunities for better peer interaction, for support, for participation in various youth engagement activities, for developing new skills and for building their social networks enabling freedom of expression and movement. Peer education conducted through the YICs showed a greater effect than peer education without a community- approved physical space for adolescents to learn and to share.

Youth Information Centres adopted a behaviour change communication strategy for empowering young unmarried and married girls and boys. Under the intervention strategies, life skill education was provided which addressed the breaking down of community barriers. These included discussion about their concerns for sexual and reproductive health such as the use of condoms and other contraceptives. Separate sessions were conducted with boys for discussing male responsibility, which led to reduction in early marriage, early pregnancy and increased school retention in the project area. The current findings are in corroboration with interventions conducted in other Indian settings (in the states of Bihar and Maharashtra) which have also used the safe spaces for adolescent clubs, sessions with family members and counseling of boys. These have also led to reduction of early marriage, early pregnancy and increased use of maternal health services such as antenatal care, post-natal care and STI/HIV services [20, 21].

Another interesting finding was that lack of access to media was linked to higher risk of early marriage. There is a wide range of empirical evidence from various studies in Asia and Africa that show that exposure to mass media plays a crucial role in preventing early marriage. Communication processes through social groups, radio and television address issues of early marriage which play a pivotal role for young girls, boys and their families in empowering them to fight against the social pressures of early marriage [22]. Additionally, community-based reproductive health agents that advocate delayed marriage from the perspective of a girl’s education and reproductive health, including strategies such as local campaigns, community conversations, school-based peer education, and public forums with religious leaders and panchayati raj institution members, to discuss the negative consequences of early marriage and childbearing, have shown an effect on reducing early marriage [23, 24].

The findings also suggest that girls and boys who came from SC/ST or other backward classes had a higher risk of early marriage. The information provided by the key stakeholders of our project showed that among the marginalized communities, lack of education, poverty, parental pressure of early marriage and lack of safety for girls are crucial factors for child marriage. There are various studies conducted in India and in neighbouring countries such as Nepal that show that coming from a particularly backward caste/class is a risk factor for early marriage, early pregnancy and sexual violence [25–27].

Additionally, in our retrospective analysis of this project, we found that the mean age of marriage of the older sibling (of the participants) was 15.83 years and the mean age of marriage of the participants after the intervention increased to 17.03 years. Therefore, we can infer that the intervention strategies adopted in the project did show an effect in reducing early marriages. Evidence from formative research has also shown the intervention for providing life skills and reproductive health education to girls in the community setting through safe spaces. It showed a significant effect for gradual decrease in the proportion of girls marrying before 18 years of age [28]. The study had a similar finding in relation to early pregnancy.

In our participants who came from the SC/ST and other backward classes had less access to mass media and had a higher risk of early pregnancy. However, the effect was diluted in the regression model after adjusting for other confounders. The speculative reason for this could be that other factors such as lack of partner communication, gender power dynamics, and/or lack of knowledge and awareness of contraceptive use, were playing a vital role for an early pregnancy. The study found that young people who came to YICs have a lower risk of early pregnancy. Therefore, it could be inferred that intervention strategies adopted such as behavioural-change communication sessions with the young people, with gatekeepers (in laws), involvement of the men, inter-personal sessions on risks and consequences of early pregnancy, skills for improving partner communication, gender norms, increasing self-efficacy for contraceptive use, and referrals, did show an effect in reducing early pregnancy.

This finding was in line with a previous research project called PRACHAR, conducted in the state of Bihar. In this study too it was found that a community level intervention using an age-segmented approach through sessions with young people, newly wed couples, key stakeholders and referrals made to the health systems, did show reduction in early pregnancy [29]. The effect of reducing early pregnancy of our project could be further asserted by our retrospective analysis where we found that age at conception of the older sibling was 17.54 years, while for the participants of this project it had increased to 18.39 years.

The findings from this project suggest that YICs comprising multi-component interventions lead to an increase in school retention for both boys and girls. Similarly, one of the intervention studies in Bangladesh, named BALIKA, involving multi-component interventions including education, gender empowerment awareness and livelihoods-skills training, showed a significant impact on school retention and reducing child marriages [30]. In our project, the peer educators in the YICs conducted sessions with parents or in-laws and explained to them the importance educating young people had on increasing school retention. The retrospective analysis of this project found that the mean years of schooling for the older sibling of the participant was 6.39 years. This had subsequently increased to 7.93 years for the participants in the project area after the intervention. In addition, our qualitative data showed that the youth gained stronger negotiation skills to convince their parents to support them continue their schooling.

The results show that access to mass media also had an association with increasing school retention. This could be due to the media sensitization that was done by the project team for the delay of early marriage and increase in school retention, and could also be due to young people’s exposure to television, radio and advertisements in the papers for national schemes for free education. The results found that coming from a backward caste also led to early dropping out of school which could be due to lack of security for girls and lack of education of the parents themselves regarding encouraging their children to remain in school. There is empirical evidence that shows that there are barriers at individual, family and community level— such as lack of supportive environment by the family, poor quality of education, early marriage and coming from deprived social groups/ classes (SC/ST) among many other obstacles [27].

The qualitative analysis found that the project played a prominent role in highlighting the issue of early marriage and early pregnancy in the intervention sites. The stakeholders echoed the relevance of the intervention. It has strengthened the programme environment by imparting greater visibility to the issue of early marriage, especially among girls. This is supported by the fact that the majority of the respondents in both villages with a YIC and those without, reported that though they were not directly involved in the project, they did know about the intervention and the issues of school retention, delaying marriage and the first pregnancy. Hence, the analysis helps to bring out the exposure to YIC as a value addition to such multi-strategy interventions.

### Strengths and limitations

To the best of our knowledge there are very few evaluated projects in India that have delivered a multi-pronged intervention that focuses on early marriage, early pregnancy and school retention. Therefore, the current findings will strengthen the evidence of a multi-component intervention to early marriage. The main strength of the study was that the survey was self-reported with no interference from parents, in-laws or their partners.

A major drawback was that this project did not have a strong baseline, to compare its results with the endline survey. Therefore, the endline is a cross-sectional study design, which does not permit a comparative analysis of baseline to endline. To mitigate this limitation, we incorporated retrospective questions based on the mean age at marriage, age of conception and school drop out of the elder sibling, which was a proxy indicator to see if the intervention made some difference through the endline survey.

Our findings may not to be generalizable to all low- and middle-income countries, but we might get similar results if this model is adapted in South Asian countries with similar socio-economic conditions. With regard to measurement error there is a possibility of a recall bias when the participants answered the questions on the older siblings’ age at marriage, age of conception and age at school dropout; confirming it with the parents assures us that the chances of that are quite low. However, the odds of a recall bias for their own behaviour may be limited since it was a young population. The responses of the survey might have been under-reported as the participants might have given socially desirable answers. We tried to limit this in our data collection and analysis by maintaining anonymity and confidentiality. In our analyses we adjusted for the intervention strategies and socio-demographic confounding factors, including those that had little impact on the associations we determined. We, therefore, feel that residual confounding would be of minor importance.

## Conclusion

The study highlights that this multi-component intervention conducted in the Youth Information Centres did have an effect in reducing early marriage, early pregnancy and in increasing school retention. Exposure of mass media messages that showed an effect on reducing early marriage and school retention through the intervention also had a significant role in affecting the behaviour of young people. This study also showcases that socio-economic factors such as coming from a lower caste play a vital role in early marriage among girls and school drop-outs among girls and boys. Therefore, we recommend that similar community-based interventions be adapted and scaled up in regions with similar socio-economic backgrounds, with the aim of reducing early marriage, early pregnancy, maternal morbidity and mortalities and in increasing school retention.

## Abbreviations

ADJ: Adjusted odds ratio
AIDS: Acquired immune deficiency syndrome
CI: Confidence Interval
HIV: Human immunodeficiency virus
IIHMR: Indian Institute of Health Management and Research
OR: Odds Ratio
SAIEVAC: South Asian Initiative to end violence against children
SC: Scheduled caste
SDG: Sustainable Development Goals
ST: Scheduled tribe
STI: Sexually transmitted infection
UP: Uttar Pradesh
YIC: Youth Information Centre
